# Linguistic labels cue biological motion perception and misperception

**DOI:** 10.1038/s41598-021-96649-1

**Published:** 2021-08-26

**Authors:** Ksenija Slivac, Alexis Hervais-Adelman, Peter Hagoort, Monique Flecken

**Affiliations:** 1 Max Planck Institute for Psycholinguistics, P.O. Box 310, 6500 AH Nijmegen, The Netherlands; 2 International Max Planck Research School for Language Sciences, Nijmegen, The Netherlands; 3 Department of Psychology, University of Zürich, Zürich, Switzerland; 4 Donders Institute for Brain, Cognition and Behavior, Radboud University Nijmegen, Nijmegen, The Netherlands; 5 Department of Linguistics, University of Amsterdam, Amsterdam, The Netherlands

**Keywords:** Language, Cognitive neuroscience, Perception, Neuroscience, Visual system, Motion detection, Psychology

## Abstract

Linguistic labels exert a particularly strong top-down influence on perception. The potency of this influence has been ascribed to their ability to evoke category-diagnostic features of concepts. In doing this, they facilitate the formation of a perceptual template concordant with those features, effectively biasing perceptual activation towards the labelled category. In this study, we employ a cueing paradigm with moving, point-light stimuli across three experiments, in order to examine how the number of biological motion features (form and kinematics) encoded in lexical cues modulates the efficacy of lexical top-down influence on perception. We find that the magnitude of lexical influence on biological motion perception rises as a function of the number of biological motion-relevant features carried by both cue and target. When lexical cues encode multiple biological motion features, this influence is robust enough to mislead participants into reporting erroneous percepts, even when a masking level yielding high performance is used.

## Introduction

Perceptual systems are susceptible to a wide variety of top-down influences^1–3^, among which linguistic labels have been found to be particularly powerful. Numerous studies have demonstrated that the presentation of a lexical cue shortly before a visual target can improve discrimination^4,5^ or detection^6–9^ of that target. This effect has been shown to persist even when the visual target is rendered imperceptible via masking techniques such as continuous flash suppression, suggesting that any observed perceptual activation in these experiments is top-down in nature and in many cases label-driven^10–12^.

Lexically mediated cueing appears to be uniquely effective in eliciting perceptual activation changes, compared to similarly informative non-linguistic cues, such as environmental sounds^5^, suggesting that linguistic labels have privileged access to a substrate relevant to perception. The *label feedback hypothesis* formalised this supposition, positing that while non-linguistic cues, such as sounds or pictures, are necessarily exemplar-bound, linguistic labels activate categorical representations by abstracting from the idiosyncrasies of individual category members and emphasising the *diagnostic features* of that category^13^. In doing so, they are able to activate a perceptual template that effectively warps the neural activation towards the labelled category^14,15^. This lexically induced perceptual activation towards the diagnostic features of the labelled category has been argued to occur in an automatic, task-independent manner, as exemplified by the fact that cueing effects on perception are reported across different types of tasks involving perceptual limens, and even when labels are task-irrelevant^16^. However, Klemfuss et al. (2012) caution against the claims that language can modulate perceptual activation, and propose an account according to which the linguistic cueing effect reflects reduced burden on working memory^17^.

However, visual perception extends beyond the commonly examined detection of static objects, which relies on contour (i.e., shape or form) recognition (for a discussion on stimulus and task complexity in cueing paradigms, see^18^). It also includes motion perception. Motion is inherently dynamic and transient, and perceiving it requires integration of relevant elements over space and time for a successful construal of a coherent percept. A small number of studies have used dynamic, point-light stimuli, such as random dot motion (RDM), to examine the influence of lexical cues on the perception of motion. Identification of RDMs’ direction of motion requires integration of the constituent elements (position and kinematics of individual dots), into a coherent, directional percept. Using RDMs set at the coherence decision limen, it has been shown that motion verbs (e.g., *rise*, *fall*) as linguistic cues bias the perception of RDMs—verbs congruent with direction of the dominant motion vector of RDMs facilitate the judgement of its principal motion direction, while incongruent verbs reduce the accuracy of such judgement^8,9^. These studies provide evidence that the perception of dynamic stimuli is susceptible to linguistic influence.

Such results raise the possibility that even more complex, dynamic point-light stimuli are subject to lexical cueing effects. One such class of stimuli is point-light figures (PLFs), used to study biological motion perception^19^. The perception of PLFs is *compositional*, in that it requires the observer to compose disconnected dots representing bodily joints and their local kinematics into a unified percept of a human figure in action (‘form-from-motion’ stimuli)^20^. The category of biological motion encompasses a wide variety of different actions performed by biological entities (e.g., for humans, *walking* or *cycling*) the perception of which requires an integration of several different features, most diagnostic (or defining) of a particular type of action being *form* and *kinematics* characteristic of that action. In the case of PLFs, these features point to different aspects of the stimulus assembly into the global percept: local information on kinematics is given by the individual dots in the earlier stages of target composition, while global form information emerges upon the successful binding of the dots into a recognisable figure^21–23^. Presenting such stimuli in combination with lexical cues conveying the notion of form and kinematics, individually or in combination, can reveal how different aspects in the process of target configuration are affected by cues encoding different features relevant to biological motion.

For example, in relation to the concept of biological motion, the word *brother* makes the feature of human form (i.e., human body) directly available to us, but carries no information about an action the named entity might be engaged in. The word *rower*, on the other hand, makes both the information about the human form (i.e., human body) and the particular type of kinematics necessary to perform such action (sitting position with a characteristic arm movement) directly available to us.

This feature-based conceptualisation of the content of linguistic labels in combination with *form-from-motion* PLF stimuli allows us to test the hypothesis that the efficacy of lexical top-down influence on perception lies in the ability of those labels to highlight and activate conceptual and perceptual representations of category-diagnostic features, and bias the perception of the visual input towards the labelled category. In this study, we therefore examine how the encoding of biological motion features—form and kinematics—in linguistic labels, modulates the strength of linguistic influence on the perception of biological motion represented in PLFs performing an action. In order to achieve this, we manipulate the number of biological features encoded in linguistic labels (no feature: no biological form or kinematics, single feature: biological form only, or multiple features: biological form and kinematics), and their degree of overlap with the PLF targets performing an action (Experiment 1), as well as congruency between label and target for cues with multiple feature availability (Experiment 2) in a biological motion detection task. To reject the possibility that any cueing effect depends solely upon visual form detection (only biological form feature encoded in the visual target), we also test whether lexical cues can affect the orientation discrimination of not only naturally moving PLFs, but also those captured in a recognizable, action-characteristic frame and stripped of the local kinematics feature, but moving coherently in a rigid manner (horizontally translating, or ‘gliding’, figures; Experiment 3).

Our view of the mechanism underlying the supposition made by the label-feedback hypothesis in the context of the current study is the following: form and (when encoded) kinematics features, when delivered lexically, co-activate perceptual form and kinematics representations relevant to the named action. This activation is not an all-or nothing phenomenon, but rather gradually becomes more extensive with the number of target relevant features encoded in the label. Cues impoverished with respect to the visual target, with only one (form-only) feature encoded, would therefore fail to evoke a comprehensive perceptual template necessary for target recognition (form *and* kinematics encoding neurons), and as such may exert only a weak influence on target perception. In other words, because the form-only, lexically induced bias is not strong, congruency will not give the perceptual system awaiting visual target a strong initial boost, but it will also allow it to ‘recover’ more quickly in the case of cue-target mismatch. In the case where lexical cues encode more features (both form and kinematics), they will engage a more comprehensive conceptual and perceptual representation, reflecting both form and kinematics encoding. By doing so they will bias the perceptual activation more strongly towards the labelled category and as a result exert a stronger influence on the perception of the incoming target in the following way: when congruent, the (pre-)activated neurons overlap with those that would need to be activated for target perception, such that they are already ‘firing’ by the time the visual input arrives (the activation is already ongoing). When incongruent, they will derail target perception, because the induced pattern of activation does not overlap with that necessary for target perception, i.e., the ongoing lexically induced activation is thus misleading with respect to the target, and needs to be corrected for successful target perception (uninformative or ‘mismatched’ template needs to be suppressed while the target matching one needs to get activated). In this featurally more comprehensive case (i.e., cues with multiple features), therefore, the congruent lexical boost will be stronger compared to that exerted by featurally impoverished (single feature) cues, but the recovery period in the case of the cue-target mismatch will also take longer or be harder to achieve within a short time period.

If feature activation drives lexical cueing effect on perception, the immediate availability of both form and kinematics features carried by lexical cues is hypothesized to exert a stronger influence on biological motion perception, compared to cues with single (form) feature availability: when congruent with the target, we expect them to amplify visual detection and interfere with rejection; when incongruent with the target, we expect them to interfere with visual detection and facilitate rejection.

In other words, we expect that the congruent biological motion cues will lead to an overall, conceptual and perceptual, bias towards the labelled category, which will result in a shift in Criterion: higher detection (hit) rate on trials with coherent PLFs, but also higher false alarm rate on trials with scrambled PLFs, with participants wrongly thinking they are seeing what has been prompted by the cue. This bias is hypothesized because even in the absence of the human form, we expect the kinematics feature preserved in the scrambled PLFs to overlap enough with the representation prompted by the cue to mislead participants into wrongly composing the scrambled PLF dots into a coherent percept. In the case of incongruence, the overlap with the target is absent when it comes to the kinematics feature, which is a particularly important clue for action recognition among the masking dots, so we expect a decrease in hit rate and an increase in correct rejection rates.

Given our experimental design and hypotheses, i.e., we expect both the target coherent distribution and the target scrambled distribution to shift as a function of our cues, we will interpret any such shifts in our results as reflecting conceptual and perceptual bias (cf. ^24^). In other words, we do not ascribe the bias induced by the cues to one single processing level. While we do think that Criterion scores can indicate the participant’s strategy (decision or response level), we echo previous accounts stating that this is not the only bias that Criterion reflects. As has been argued before^24–27^, we recognise the necessity to interpret signal detection theory indices in line with the experimental design when conducting psychological detection or discrimination experiments. The abundant evidence showing that linguistic top-down influences can and do regularly modify conceptual and perceptual alongside higher level decision processes^14,28,29^, and that those modifications affect Criterion scores ^27,30^ further justifies our claim that the bias observed here should not be restricted only to the decision making level, but also encompasses conceptual and perceptual level.

Finding that a lexical cueing effect is modulated by the overlap in the amount of features encoded in lexical cues and visual targets would be the first empirical demonstration of a process of feature activation as underlying such linguistic influences on perception.

## Methods and results

### Experiment 1

We examine whether lexical cues carrying and overlapping in multiple features diagnostic of biological motion category (form and kinematics) exert a stronger effect on the PLF detection, compared to single-feature overlap (form-only) conveying cues, as well as no-feature overlap (general motion cues, that had overlapping features with the mask rather than the target; see details below).

### Methods

#### Participants

Fifty-one native speakers of Dutch (43 female, 8 male; mean age: 23.56; age range: 19–33) recruited from the Max Planck Institute (MPI) participant database took part in the experiment. Eleven participants failed to reach the inclusion criterion during the thresholding procedure (see below) and were therefore excluded from the analysis, resulting in 40 complete datasets (33 female, 7 male participants; mean age: 23.38; age range 19–33). All participants were right-handed and had normal or corrected-to-normal vision, and no reading difficulties. All the participants gave their informed consent and received financial compensation for their participation. All the studies presented in this article were approved by the Ethics Board of the Social Sciences Faculty of Radboud University (ESCW). All experiments were carried out in accordance with the recommendations of the seventh revision of the Declaration of Helsinki (2013) regarding participants’ informed consent.

#### Stimuli

All stimuli were generated using the Psychophysics Toolbox^31^ within MATLAB R2016a (MathWorks, Natick, MA). Both lexical cues and visual targets were presented in white (luminance: 160 cd/m^2^) on a grey background (luminance: 37 cd/m^2^).

The lexical cues were presented in Dutch and consisted of three lexical cue categories with 4 nouns each, and one control (no language) cue category, the string #### (see Table S1). The three categories of lexical cues encompassed two categories semantically congruent with the target, and one semantically incongruent category. The former contained biological motion cues, conveying both biological form and kinematics information (e.g., *rower, walker*), and biological form cues, with biological form but not motion information (e.g., *brother, father*). Semantically incongruent cues were general motion words (e.g., *snow, smoke*), which matched the directionality of the masking RDM dots on every trial (e.g., *snow*—downward motion, *smoke*—upward motion) rather than the PLF target.

The visual targets consisted of 13 white dots comprising a point-light figure (PLF; size: 3.59–4.36 cm (horizontal) × 5.95–6.57 cm (vertical); speed: 30 frames/s) embedded in a random dot motion mask (RDM), with circular aperture (22 × 23.5 cm; number of dots: 866; dot size: 0.528 × 0.528 mm, dot motion speed: 0.528 mm/frame, dot lifetime: 10 frames, at 30 frames/s), presented in the middle of the screen. Four PLF types, performing four types of actions: wood-cutting (with an axe), walking, rowing and dancing, were selected from an action database^32^. The PLF we labelled and introduced to participants as ‘dancer’ was originally labelled as ‘waving’ by the authors of the database, but was described by participants in the original study as ‘dancing’ (cf. ^32^). The most important criteria for the action selection were that they involved whole-body movement, i.e., all the dots representing major joints were in motion, and that they could be easily expressed by a single noun. On every trial, PLFs were presented, facing to the left or to the right in sagittal view (90°), in the centre of the RDM aperture. They were shown either in their coherent form, comprising a human figure in action (target present condition), or in a scrambled form, where the initial locations of the landmark dots were randomly positioned within the perimeter of the coherent PLF, while their individual kinematics were preserved. This manipulation renders the target unrecognizable as a coherent biological figure in motion (target absent condition).

On every trial, a coherent or scrambled PLF was embedded in an RDM mask. The masking RDM dots were identical to the target PLF dots in size and luminance, but their kinematics were different. On every trial, a certain proportion of RDM dots (see below) moved coherently in an upward or downward direction, while the rest (i.e., incoherent dots) were re-drawn in a random location at every monitor refresh. Piloting of this masking technique showed that masking efficacy increased with decreasing coherence.

Individual masking levels (i.e., the percentage of RDM dots surrounding the PLF target, moving coherently in an upward or downward direction) were determined for each of the four PLF types and per participant using a Bayesian adaptive staircase procedure (QUEST^33^). For every action, the threshold was collapsed across upward and downward RDM motion direction as well as across left and right PLF orientation. At the end of the staircase procedure (96 trials per action), we extracted four thresholds for every participant, which reflected the masking level at which the four actions yielded approximately 75% accuracy on a biological motion detection task (see below). Participants who did not reach the 75% accuracy performance on all four actions even when all the noise dots were moving coherently (i.e., at the easiest level of target detection) during the thresholding procedure were excluded from the experiment.

#### Procedure

Participants were seated in a dimly lit room, approximately 60 cm away from the monitor. Stimuli were displayed on an Acer monitor (17″, 1280 × 1024, 60 Hz refresh rate). The participants received both spoken and written instructions (on the screen) prior to doing the experiment.

The experiment consisted of three parts: familiarization, practice and thresholding, and cueing experiment, all described in detail below.

##### Familiarization

The experiment started with a short familiarization session, during which all the visual targets and lexical cues were presented to the participants, with the instruction to carefully observe the stimuli. The presentation of the PLFs was accompanied by a one sentence description of the type of action they engaged in, e.g., “Je ziet zometeen een figuur, die wandelt” (“*You will see a walking figure*”).

##### Practice and thresholding

**Figure 1:**
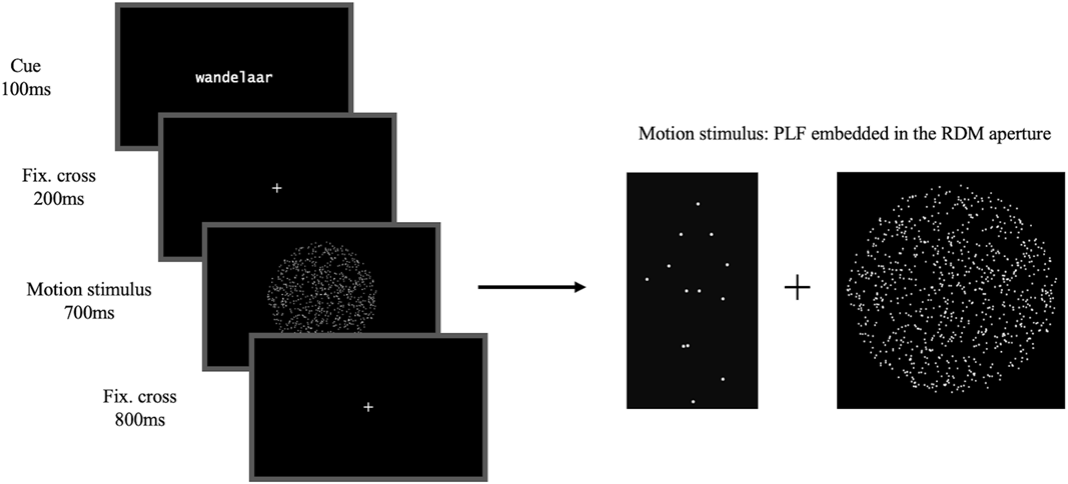
Trial design. A string cue is displayed at the beginning of every trial, before the target. The visual motion stimulus, comprised of a PLF figure (coherent or scrambled) embedded in an RDM aperture (upward or downward moving dots at a predetermined level of coherence), is presented in the middle of the screen. Participants have 1.5 s to respond—700 ms (motion stimulus) + 800 ms (fixation cross)—with the instruction to press the button, answering whether they saw coherent biological motion yes or no, as soon as possible.

The practice session consisted of one block (128 trials) and was shortly followed by the thresholding session of three blocks (128 trials per block). Participants were instructed to monitor the screen and to indicate on each trial whether they detected coherent biological motion or not (“Do you see coherent biological motion, yes or no?”), as quickly and accurately as possible. Participants responded on a button box with left or right index fingers. The trial structure of the thresholding procedure follows that of the cueing experiment, illustrated in Fig. 1, but in order to obtain non-biased estimates of PLF detection, cues were not presented.

##### Cueing experiment

The cueing part of the experiment consisted of 4 blocks, 128 trials each (512 trials in total), and had the same task and trial structure as the thresholding session (Fig. 1). The only difference was that in this part, participants were presented with either no language cues or lexical cues prior to the visual target, following the design of the experiment. The cues were presented in the centre of the screen (font style: ‘lucidatypewriter’, font size 18). Trial presentation order was fully randomised.

#### Analysis

Data analysis was performed on 40 complete datasets. Prior to the analyses, trials with reaction times (RTs) 2.5 SD or more from the grand mean were excluded (trials with RTs above 1345.84 ms and below 427.69 ms). This resulted in the exclusion of 460 out of 20,480 trials (2.2% of trials).

##### Accuracy and RTs

We were interested in how the lexical cue categories (biological motion, biological form and general motion) influenced both the detection of coherent and rejection of scrambled PLFs. We expected the cues that exert a facilitatory effect on the detection of a coherent target to also be detrimental to the rejection of scrambled targets (i.e., would lead to a higher false alarm rate) and vice versa. First, we compared the three lexical cue categories to the control (no language) cue category, for coherent and scrambled PLF conditions respectively. Further, we aimed to examine differences in the magnitude of the lexical cueing effect as a function of the number of features shared between lexical cue and target, and thus compared the lexical cue categories with one another, again separately for coherent and scrambled PLFs, in a post hoc analysis.

A Bayesian approach allows us to quantify uncertainty in relation to our findings by means of obtaining probability distributions for our parameters of interest rather than a single point estimate, as with frequentist analyses. Further, the three experiments reported here build upon each other, both theoretically and empirically, allowing us to specify priors for each analysis based on the results from the previous experiment. Therefore, we ran Bayesian linear mixed effects models, as implemented in the R package brms^34,35^. Post hoc analyses were conducted with the R package emmeans^36^.

Both accuracy (Bernoulli distribution, logit link) and RT (Gaussian distribution, identity link, with log-transformed RTs) models were fitted with the maximal, hypothesis-driven, non-singular structure supported by the data^37–39^. The resulting model consisted of the predictor ‘cue category’ (4 levels: biological motion, biological form, general motion and no language) nested under the predictor ‘PLF coherence’ (2 levels: coherent, scrambled) as fixed effects, and by-subject and by-item random intercepts and slopes for PLF coherence as random effects. For fixed effects, we used simple effect coding, with the PLF coherence predictor coded as (coherent: 0.5, scrambled: − 0.5), and the cue category predictor coded as (no language cues were base coded as − 0.25, contrasting condition of each column as 0.75).

Bayesian models were run with informative priors appropriate for the effects expected in this study. Priors for both accuracy and RT models’ contrasts of interest were taken from the estimates and 95% credible intervals of the posteriors of pilot data from three participants. Prior sensitivity analyses were performed to ensure the choice of priors did not influence our results. Both models were fit with 4 chains, 10,000 iterations each, 3000 of which were the warm-up phase, with a thinning factor of 3. To assess correct convergence, we verified that there were no divergent transitions, R-hats were all equal to one, the number of effective samples were at least 10% of post-warmup samples, and chains were visually inspected for stationarity.

##### Signal detection analysis (Criterion and d′)

In order to separate participants’ conceptual, perceptual and decision biases from perceptual sensitivity/discriminability, we calculated Criterion and d′ scores for our four cue categories per participant. These values were computed based on z-transformed scores of participants’ hit rates (correctly reporting the presence of a coherent PLF) and false alarm rates (FA; incorrectly reporting the presence of a coherent PLF when the target was scrambled and therefore non-existent). Both scores were further compared across the four cueing conditions at the group level using unequal variance Bayesian mixed models (Gaussian distribution), with cue category as a fixed effect and by-Subject intercept as a random effect. The fixed effect had the same contrast coding scheme as in the accuracy model, and a lightly regularizing, normally distributed prior with mean of 0 and standard deviation of 2.

### Results

#### Accuracy

**Figure 2:**
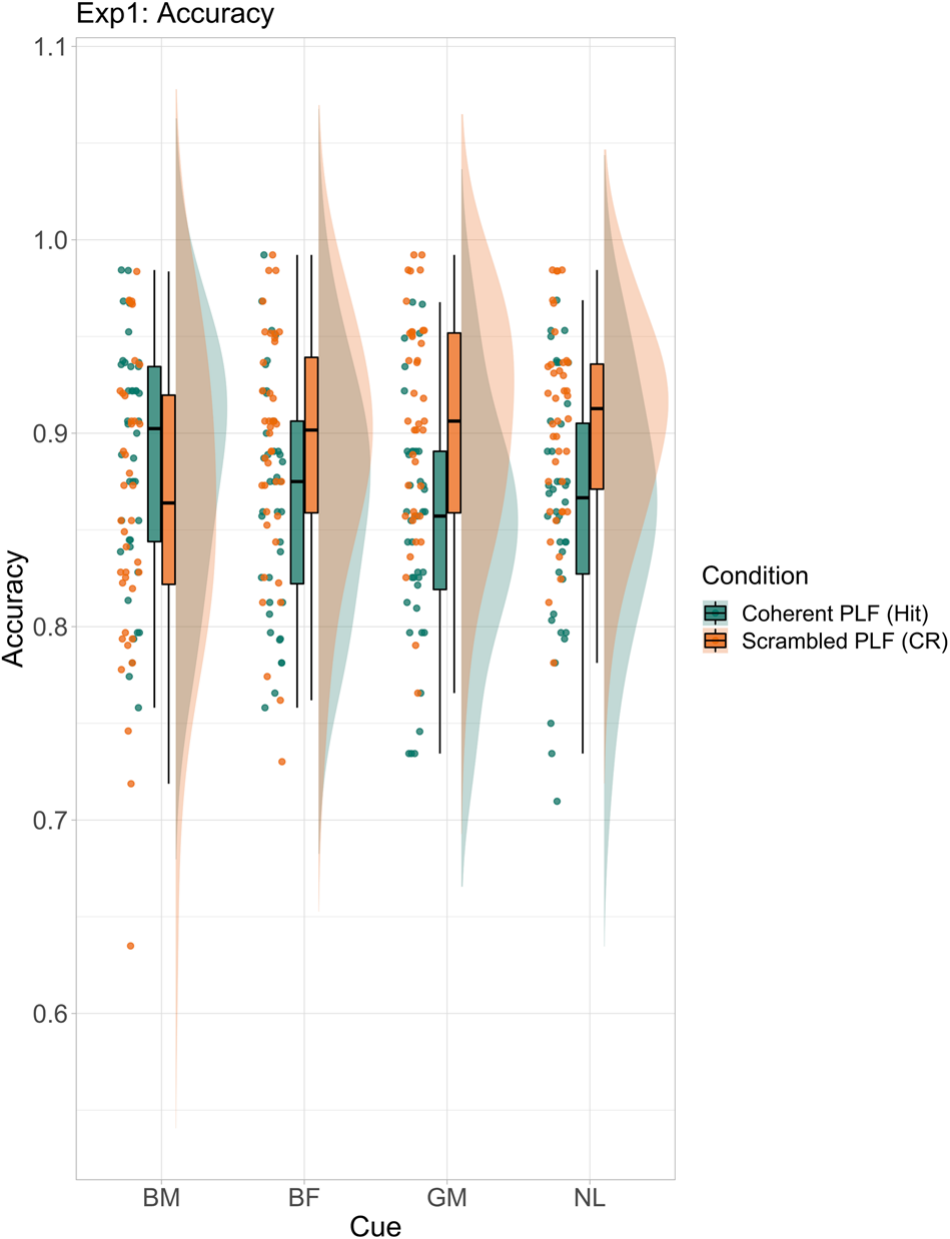
Accuracy (raw data) for coherent (hit) and scrambled (correct rejection; CR) PLF conditions for Experiment 1. Cue names are as follows: *BM* biological motion, *BF* biological form, *GM* general motion, *NL* no language.

For coherent PLFs (target present condition; mean accuracy: 86.68%; see Fig. 2), the accuracy model revealed higher PLF detection hit rate for biological motion cues, compared to each of the other three cue categories (see Table 1). The hit rate advantage for biological motion cues was found to be biggest compared to general motion cues, followed by no language cues, and was smallest compared to biological form cues (see Supplementary Fig. S1).

**Table 1:** Accuracy and RT results for Experiment 1.

	Accuracy Coherent PLF	Accuracy Scrambled PLF	log(RT) Coherent PLF	log(RT) Scrambled PLF
Contrasts	Estimate (95% CrI)	Estimate (95% CrI)	Estimate (95% CrI)	Estimate (95% CrI)
BM vs. NL	**0.23 (0.06 to 0.41)**	− **0.44 (**− **0.62 to **− **0.26)**	− **0.02 (**− **0.03 to **− **0.01)**	− 0.00 (− 0.01 to 0.01)
BF vs. NL	0.05 (− 0.11 to 0.22)	− 0.15 (− 0.34 to 0.04)	− **0.01 (**− **0.02 to **− **0.00)**	− **0.01 (**− **0.02 to **− **0.00)**
GM vs. NL	− 0.09 (− 0.26 to 0.07)	0.04 (− 0.16 to 0.23)	− 0.00 (− 0.01 to 0.01)	− **0.01 (**− **0.02 to **− **0.00)**
BM vs. BF	**0.18 (0.01 to 0.35)**	− **0.29 (**− **0.46 to **− **0.12)**	− **0.01 (**− **0.02 to **− **0.00)**	0.01 (− 0.00 to 0.02)
BM vs. GM	**0.33 (0.16 to 0.50)**	− **0.48 (**− **0.66 to **− **0.29)**	− **0.02 (**− **0.03 to **− **0.01)**	**0.01 (0.00 to 0.02)**
BF vs. GM	0.15 (− 0.02 to 0.31)	− 0.19 (− 0.38 to 0.01)	− 0.01 (− 0.02 to 0.00)	0.00 (− 0.01 to 0.01)

Cue names are as follows: *BM* biological motion, *BF* biological form, *GM* general motion, *NL* no language. Bolded are estimates and credible intervals that did not cross zero.

For scrambled PLFs (target absent condition; mean accuracy: 89.17%; see Fig. 2), the model showed the highest false alarm rate (lowest correct rejection) on trials with biological motion cues compared to the other three cue categories respectively (see Table 1). This difference was again biggest when biological motion cues were contrasted with general motion cues, followed by no language cues, and smallest compared to biological form cues (see Supplementary Fig. S1).

#### Criterion and d′

Criterion differed across cue categories as follows: biological motion vs. no language (estimate = − 0.17, 95% CrI = − 0.25 to − 0.10); biological motion vs. biological form (estimate = − 0.12, 95% CrI = − 0.20 to − 0.04); biological motion vs. general motion (estimate = − 0.22, 95% CrI = − 0.29 to − 0.15); biological form vs. general motion (estimate = − 0.1, 95% CrI = − 0.17 to − 0.03). Sensitivity (d′) did not vary as a function of cue category. These results show that participants were biased towards reporting coherent biological motion (more liberal with their ‘yes’ answers) when cued by biological motion words, compared to the other three cue categories (see Supplementary Table S2).

#### RTs

**Figure 3:**
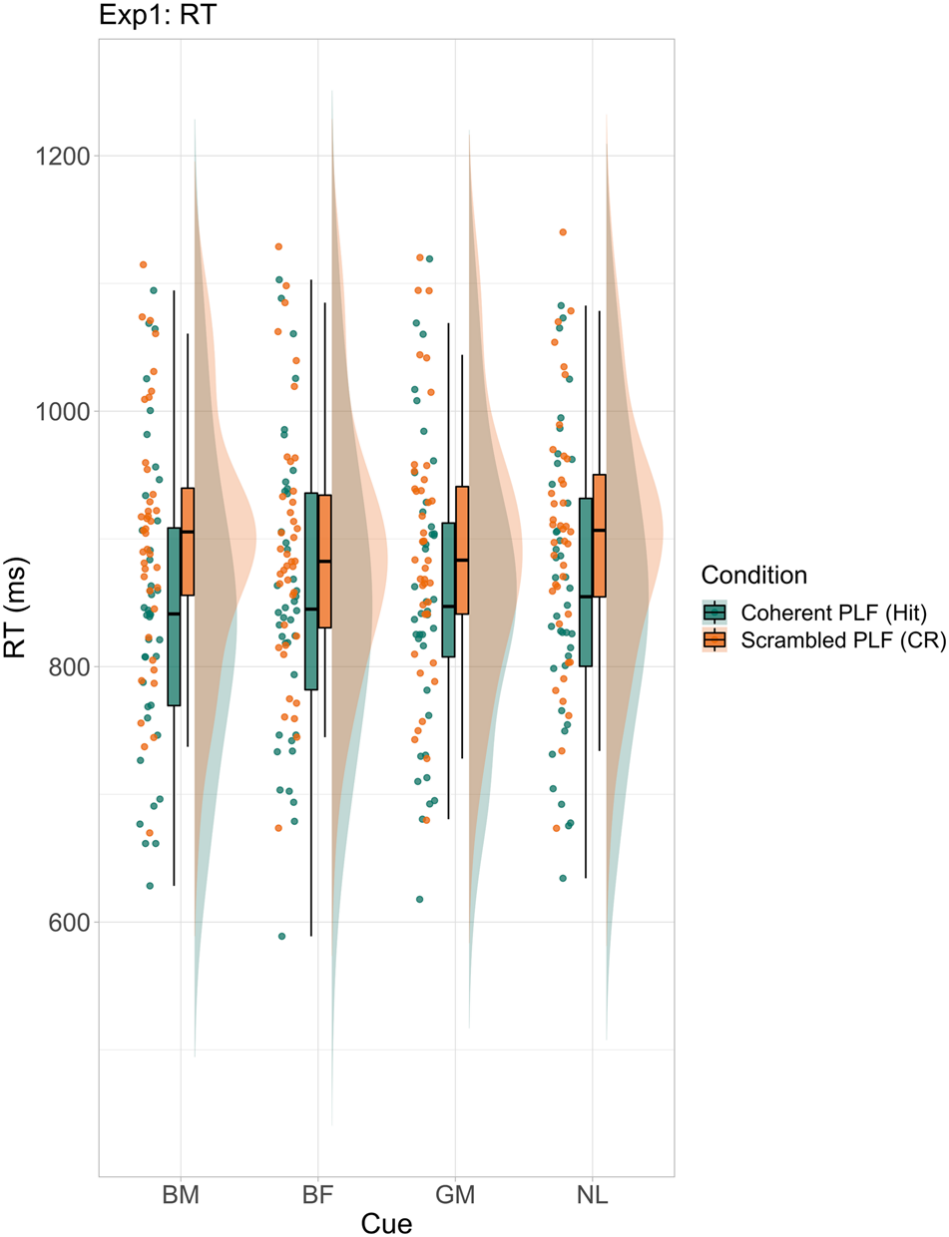
RTs (raw data) for coherent and PLF conditions for Experiment 1. Cue names are as follows: *BM* biological motion, *BF* biological form, *GM* general motion, *NL* no language.

For coherent PLFs (target present; mean RT: 852.89 ms; see Fig. 3) the model revealed fastest RTs for biological motion cues compared to each of the other three cue categories (see Table 1). Furthermore, biological form cues led to faster RTs compared to no language cues. The RTs for biological motion cues were fastest compared to general motion and no language cues, while that difference was smallest compared to biological form cues (see Supplementary Fig. S2).

For scrambled PLFs (target absent; mean RT: 896.86 ms; see Fig. 3), the model showed slowest RTs for biological motion cues compared to the general motion cues (see Table 1). Furthermore, both biological form and general motion cues led to faster RTs compared to no language cues (see Supplementary Fig. S2).

### Experiment 2

Experiment 1 showed that cues encoding multiple biological motion features enhanced coherent PLF detection and interfered with scrambled PLF correct rejection. However, all biological motion trials included congruent cue-target pairs. We thus cannot rule out that the observed effect was due to this cue-target contingency. The finding that congruent cues facilitate coherent PLF detection also raises the question of whether incongruent cues (i.e., cues with both form and kinematics features encoded but not congruent with the PLF target) may lead to equally strong detrimental effects. We therefore extended our investigation by incorporating incongruent biological motion, with the human form feature congruent and kinematics feature incongruent with the target, cue-target pairs into the paradigm.

### Methods

#### Participants

Fifty-five native Dutch speakers (45 female, 10 male, mean age: 23.56, age range: 19–33) recruited from the MPI participant database took part in the experiment. Fifteen participants failed to reach the inclusion criterion during the thresholding procedure and were excluded from the analysis, resulting in 40 complete datasets (31 female, 9 male, mean age: 23.58, age range: 19–33). All participants were right-handed and had normal or corrected-to-normal vision, and no reading difficulties. All the participants gave their informed consent and received monetary compensation for their participation.

#### Stimuli

The same stimuli and procedure were used as in Experiment 1, with the following changes. The four PLFs were slightly rotated from 90° to 45° left and right facing profile, to create a more visible form angle, i.e., to minimize the potential crossing of the landmark dots. For linguistic cues, the biological form cue category was replaced with incongruent biological motion cues (e.g., ‘dancer’ followed by the ‘walker’ target), in order to test the effect of congruency within the biological motion category on PLF detection. Every cue was paired with each PLF stimulus, resulting in two incongruent pairings (general motion and incongruent biological motion) and one congruent pairing (congruent biological motion).

#### Procedure

The experiment had the same parts as Experiment 1, but this time after the familiarization section, participants went through 4 practice blocks before proceeding to the thresholding procedure. The additional practice block was added to increase participant familiarity with the stimuli, because mean accuracy in Experiment 1 was well above the level that had been estimated during the thresholding procedure (75%), suggesting that some learning may have taken place after the practice block and during the thresholding procedure. Stimulus presentation order in all parts of the experiment was again fully randomised.

#### Analysis

Data analysis was performed on 40 complete datasets. Prior to the analyses, trials with RTs 2.5 SD or higher from the grand mean were excluded (trials with RTs above 1367.145 and below 441.7752). This resulted in the removal of 528 out of 20,480 trials (2.5% of trials).

Bayesian linear mixed effects models for the accuracy, RT, Criterion and d′ analyses had the same fixed/random effects structure and coding scheme as Experiment 1. Priors (Gaussian distribution) for both accuracy and RT models contrasts of interest were taken from the posterior estimates and 95% credible intervals from Experiment 1 and differed across conditions in line with the estimates and credible intervals in Experiment 1. Priors for Criterion and d′ were again lightly regularizing, as in Experiment 1.

### Results

#### Accuracy

**Figure 4:**
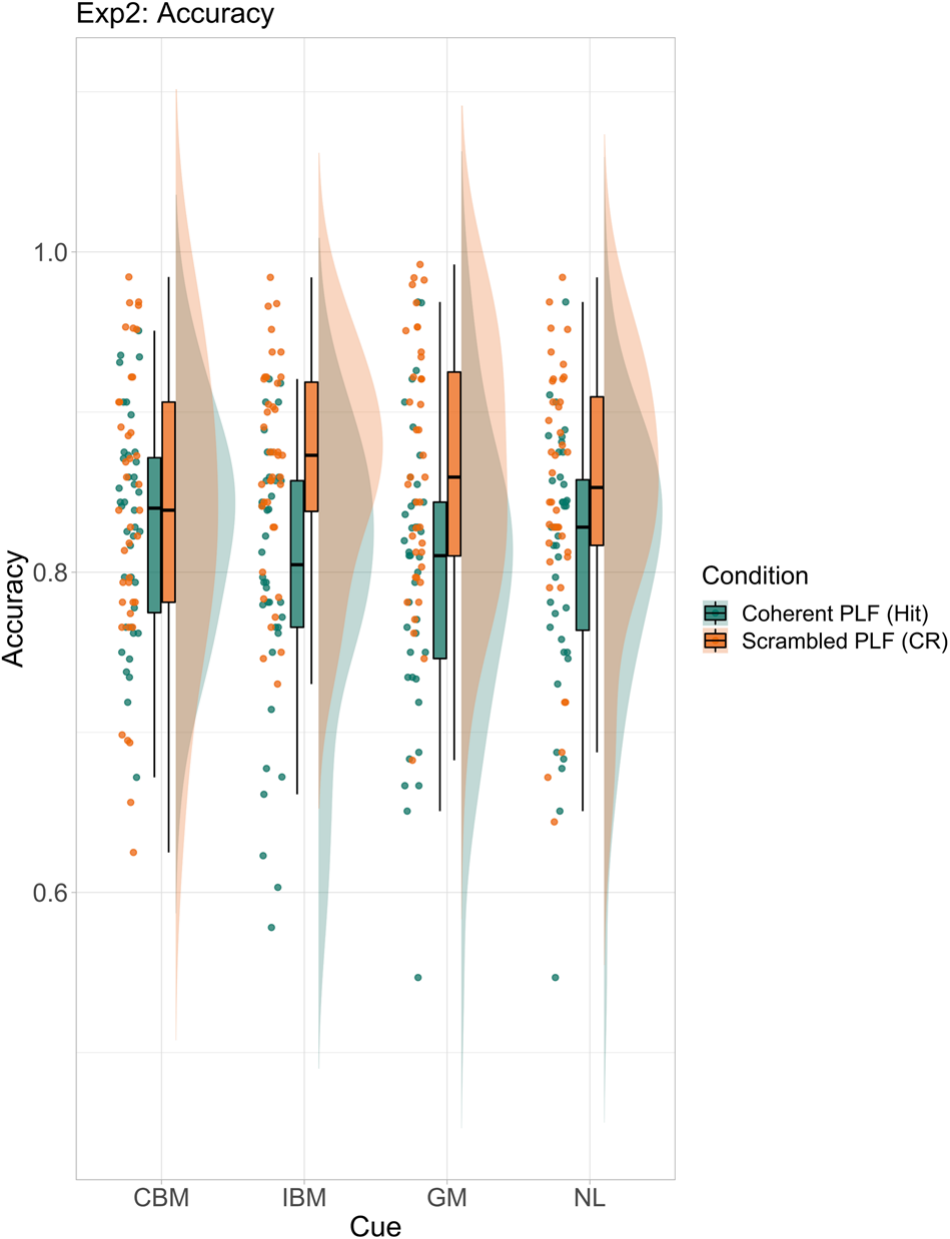
Accuracy (raw data) for coherent (hit) and scrambled (correct rejection; CR) PLF conditions for Experiment 2. Cue names are as follows: *CBM* congruent biological motion, *IBM* incongruent biological motion, *GM* general motion, *NL* no language.

For coherent PLFs (target present; mean accuracy: 80.78%; see Fig. 4), the model revealed a higher PLF detection hit rate for congruent biological motion cues compared to the other three cue categories respectively (see Table 2). This difference was larger compared to semantically invalid (general motion and incongruent biological motion) cues than for no language cues (see Supplementary Fig. S3).

**Table 2:** Accuracy and RT results for Experiment 2.

	Accuracy Coherent PLF	Accuracy Scrambled PLF	log(RT) Coherent PLF	log(RT) ScrambledPLF
Contrasts	Estimate (95% CrI)	Estimate (95% CrI)	Estimate (95% CrI)	Estimate (95% CrI)
CBM vs. NL	**0.15 (0.01 to 0.30)**	− 0.12 (− 0.28 to 0.03)	− **0.02 (**− **0.03 to **− **0.01)**	0.00 (− 0.01 to 0.01)
GM vs. NL	− 0.07 (− 0.21 to 0.07)	0.13 (− 0.03 to 0.28)	− 0.01 (− 0.01 to 0.00)	− 0.00 (− 0.01 to 0.01)
IBM vs. NL	− 0.06 (− 0.21 to 0.08)	0.13 (− 0.02 to 0.29)	0.00 (− 0.00 to 0.01)	− 0.00 (− 0.01 to 0.01)
CBM vs. GM	**0.22 (0.08 to 0.37)**	− **0.25 (**− **0.41 to **− **0.09)**	− **0.02 (**− **0.03 to **− **0.01)**	0.00 (− 0.01 to 0.01)
CBM vs. IBM	**0.22 (0.07 to 0.36)**	− **0.26 (**− **0.42 to **− **0.1)**	− **0.03 (**− **0.04 to **− **0.02)**	0.01 (− 0.00 to 0.01)
GM vs. IBM	− 0.01 (− 0.15 to 0.13)	− 0.01 (− 0.17 to 0.16)	− **0.01 (**− **0.02 to **− **0.00)**	0.00 (− 0.01 to 0.01)

Cue names are as follows: *CBM* congruent biological motion, *GM* general motion, *IBM* incongruent biological motion, *NL* no language. Bolded are estimates and credible intervals that did not cross zero.

For scrambled PLFs (target absent; mean accuracy: 89.17%; see Fig. 4), the model showed the highest false alarm rate (lowest correct rejections) for biological motion cues compared the two lexical cue categories respectively (see Supplementary Fig. S3).

#### Criterion and d′

Criterion differed as a function of cue category as follows: congruent biological motion vs. general motion (estimate = − 0.12, 95% CrI = − 0.2 to − 0.04); congruent biological motion vs. incongruent biological motion (estimate = − 0.11, 95% CrI = − 0.18 to − 0.03). Sensitivity (d′) did not vary as a function of cue category. The results indicate that participants were less conservative (less likely to report target as absent) with their answers when cued by congruent biological motion words than other lexical cues (see Supplementary Table S2).

#### RTs

**Figure 5:**
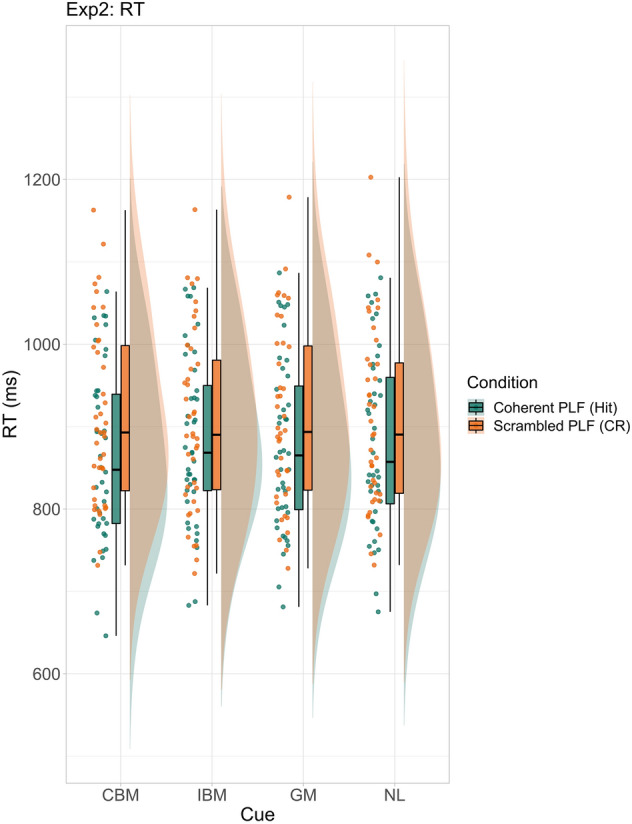
RTs (raw data) for coherent and PLF conditions for Experiment 2. Cue names are as follows: *CBM* congruent biological motion, *IBM* incongruent biological motion, *GM* general motion, *NL* no language.

For coherent PLFs (target present; mean RT: 873.97 ms; see Fig. 5) the model revealed fastest RTs for congruent biological motion cues compared to other three cue categories respectively (Table 2). Furthermore, general motion cues led to faster RTs than incongruent biological motion cues, suggesting that incongruent biological motion cues were most detrimental to the task (see Supplementary Fig. S4).

For scrambled PLFs (target absent; mean RT: 908.31 ms, see Fig. 5), the model did not reveal any notable differences between cue categories (see Supplementary Fig. S4).

### Experiment 3

Experiments 1 and 2 showed that lexical cues encoding multiple biological motion features (both form and kinematics) had the strongest influence on biological motion detection. However, feature encoding in the previous two experiments was manipulated only for lexical cues, while PLF targets always contained both form and kinematics features. We therefore could not dismiss the possibility that while both form and kinematics features do need to be represented by linguistic labels, form feature alone conveyed by the visual target might be enough for the lexical cueing effect on perception to be observed.

In Experiment 3, we aimed to test whether lexical cues have an effect only on the final target form, arising from the point-light dots configuration, or if they are instead guiding the compositional process reliant on multiple diagnostic features (i.e., kinematics of the dots as well as their placement) of biological motion. For that reason, in addition to the naturally moving PLFs, we included a condition with visually absent biological kinematics, but present biological form (PLFs frozen in a canonical stance and translating horizontally in space; ‘gliders’).

### Methods

#### Participants

Sixty native Dutch speakers (47 female, 13 male, mean age: 23.37, age range: 20–33) recruited from the MPI participant database took part in the experiment. Twenty participants failed to reach the inclusion criterion during the thresholding procedure and were therefore excluded from the analysis, resulting in 40 complete datasets (32 female, 8 male, mean age: 23.5, age range: 20–33). All participants were right-handed and had normal or corrected-to-normal vision, and no reading difficulties. All participants gave their informed consent and received monetary compensation for their participation.

#### Stimuli

Experiment 3 had the same experiment and trial structure as Experiment 2, with some modifications. Lexical cues remained the same as in Experiment 2, but along with the naturally moving PLFs (‘naturals’; presented in Experiment 1 and 2) an additional type of motion—gliding PLFs (‘gliders’, speed: 0.528 mm/frame)—was introduced as a visual target on 50% of the trials in an event related manner. Gliders were PLF animations captured in a recognizable frame and made to translate rigidly back and forth in space inside of the RDM aperture, at the speed that approximately matched the speed of the RDM dots. This manipulation made it possible for the PLFs to retain form in the absence of local kinematics, thus allowing for evaluation of the cueing magnitude of the form-only visual target.

The addition of gliders required a change of task: the figures were presented in either their upright (normal) form or inverted (upside down) form. Inverting the PLFs was expected to impair the successful composition of the dots into a resolved, canonical human form, and thereby to have a similar effect on biological motion detection as the scrambling in our Experiments 1 and 2^40^. Participants were therefore asked to discriminate between upright and inverted PLFs, placing the focus on the biological form rather than the motion itself, and making the task therefore less directly related to the lexical cues.

#### Procedure

Similar to the previous two experiments, Experiment 3 started with a short familiarization section, succeeded by practice and thresholding. This comprised two practice blocks and four thresholding blocks, 128 fully randomised trials each. Trials had the same structure as the previous two experiments. Thresholding was carried out individually for naturally moving and gliding PLFs, resulting in 8 thresholds per participant extracted after 64 trials per action and motion type, but was otherwise identical to the previous two experiments. The cueing experiment consisted of 4 blocks, 256 fully randomised trials each (1024 trials in total, 512 trials per motion type).

#### Analysis

Forty complete datasets were analysed. Prior to the analysis, trials with RTs 2.5 SD or higher from the grand mean were excluded (trials with RTs above 1339.734 and below 350.2306). This resulted in the removal of 1519 out of 40,960 trials (3.7% of trials excluded).

For accuracies, RTs, criterion and d′, the models had a different fixed and random effects structure from Experiment 1 and 2 due to the extra ‘PLF motion type’ factor (gliding or naturally moving PLFs, labelled gliders and naturals, respectively). For accuracy and RTs, the fixed effects structure therefore included the predictor ‘cue category’ (congruent biological motion, general motion, incongruent biological motion and no language) nested under ‘PLF orientation’ predictor (upright, inverted) nested under ‘PLF motion type’ predictor (naturals, gliders). This structure allowed evaluation of the effect of lexical cues for each motion type and PLF orientation type. Random effects structure consisted of by-subject and by-item random intercepts and slopes for PLF motion and PLF coherence but not their interaction. For fixed effects, the PLF motion predictor was coded as (naturals: 0.5, gliders: − 0.5), and the PLF coherence and cue category predictors was coded as in Experiment 2. Priors for both accuracy and RT models were taken from the posterior estimates and 95% credible intervals from Experiment 2, while signal detection indices (Criterion and d′) were analysed for naturals and gliders respectively in the same way as in the Experiment 2.

### Results

**Figure 6:**
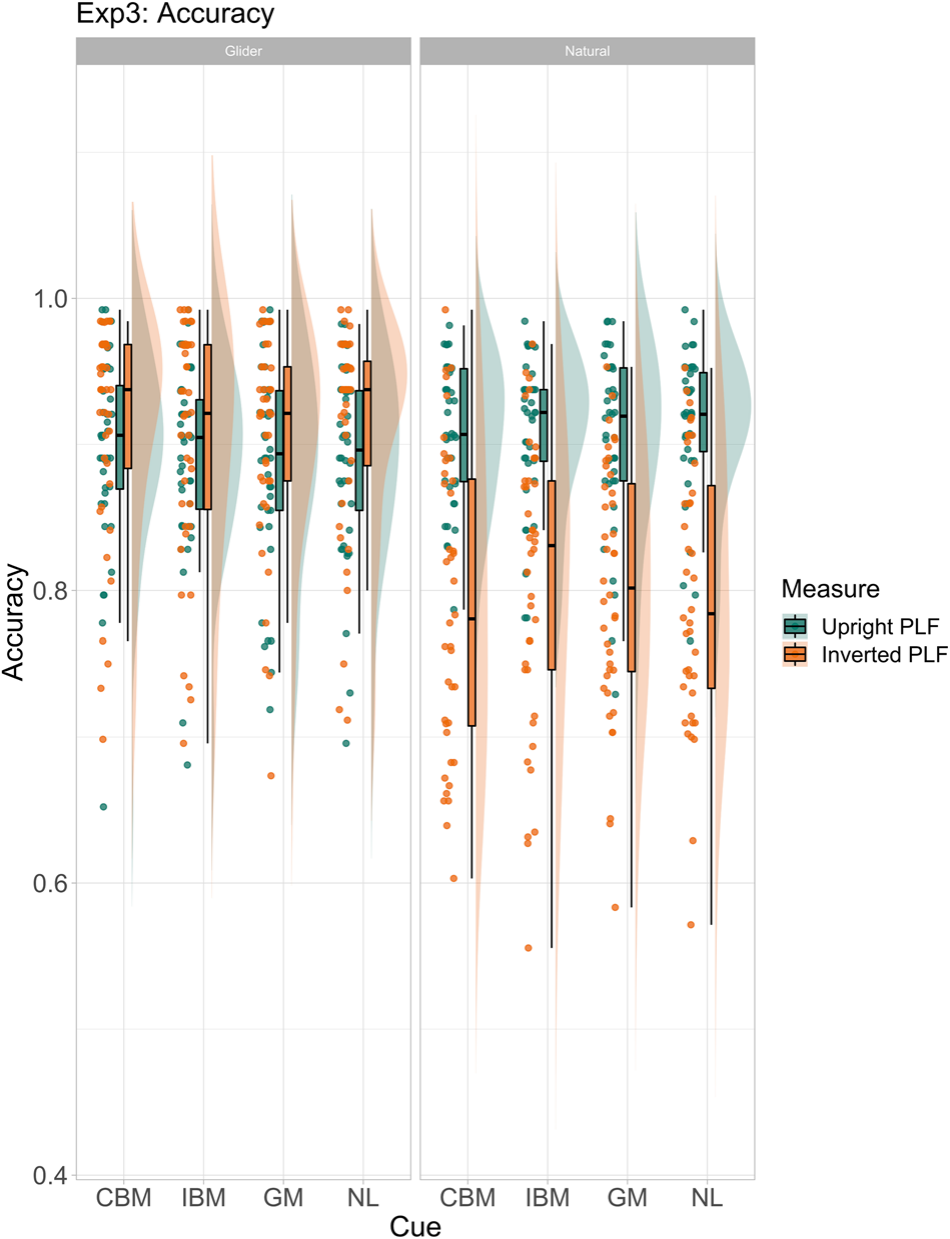
Accuracy (raw data) for glider and natural PLF motion conditions, for upright and inverted PLF orientation conditions for Experiment 3. Cue names are as follows: *CBM* congruent biological motion, *IBM* incongruent biological motion, *GM* general motion, *NL* no language.

**Figure 7:**
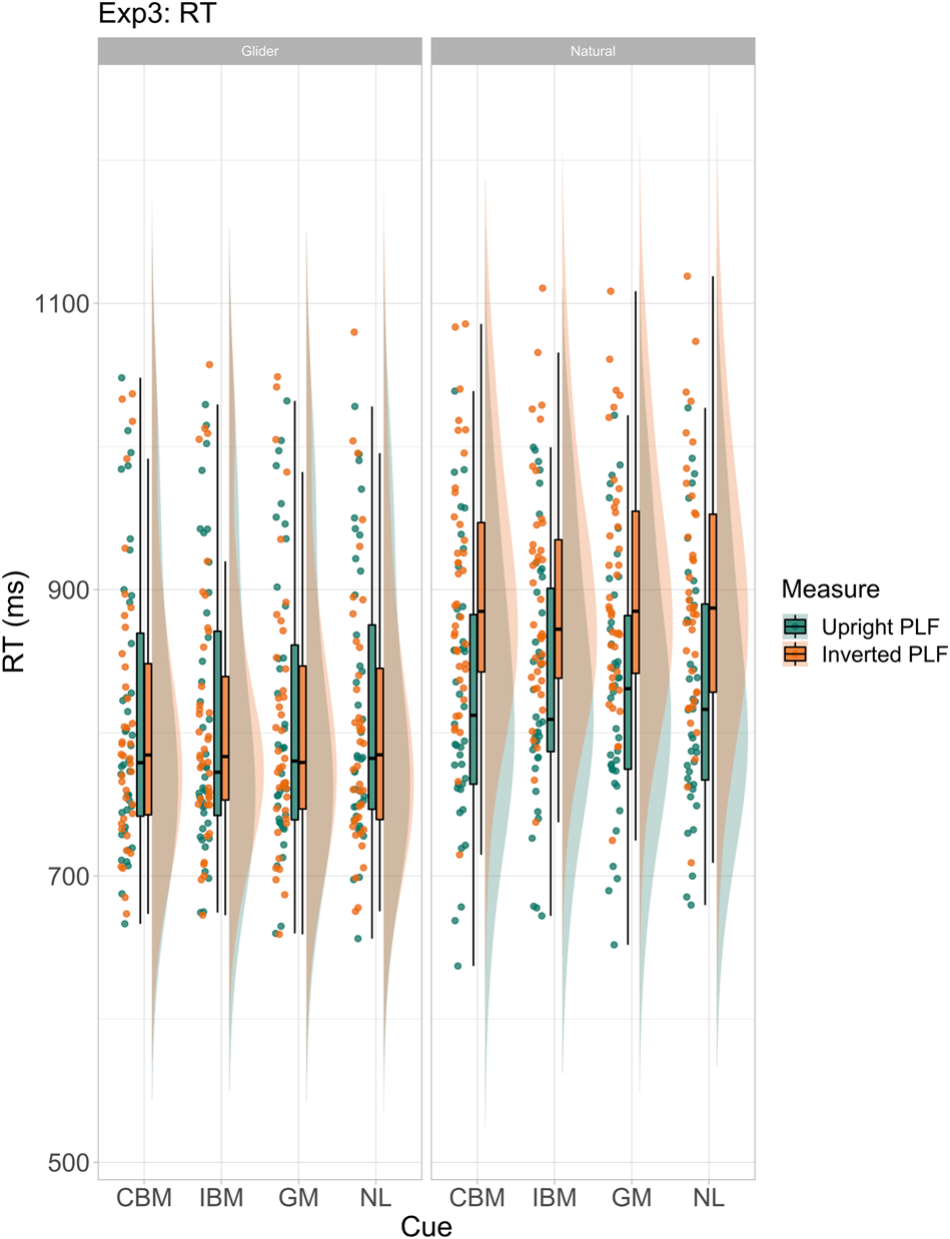
RTs (raw data) for glider and natural PLF motion conditions, for upright and inverted PLF orientation conditions for Experiment 3. Cue names are as follows: *CBM* congruent biological motion, *IBM* incongruent biological motion, *GM* general motion, *NL* no language.

Both accuracy and RT analyses (mean accuracy: 90.10%, see Fig. 6 for accuracy; mean RT: 806.84 ms; see Fig. 7 for RT) showed no discernible effects for any of the cueing categories on the discrimination of the gliding PLFs.

For naturally moving PLFs (‘naturals’, mean accuracy: 85.46%), accuracy results showed a higher false alarm rate for inverted PLFs when cued by congruent biological motion cues compared to the incongruent ones (estimate = − 0.15, 95% CrI = − 0.29 to − 0.01; see Supplementary Fig. S5). The models revealed no differences for either Criterion or d′ as a function of cue categories (see Supplementary Table S3).

RT results (‘naturals’; mean RT: 859.81 ms) revealed a faster detection rate for naturally moving upright PLFs when cued by congruent biological motion compared to incongruent cues (estimate = − 0.01, 95% CrI = − 0.02 to − 0.002; see Supplementary Fig. S6).

## General discussion

The results presented here show that perception of point-light figures is susceptible to lexical influence. Throughout the three experiments reported here, our results consistently show that lexical cues do not increase perceptual discriminability between coherent and scrambled PLFs (as reflected in the d′ scores), but rather bias the PLF detection towards the cued category (as reflected in the Criterion scores). Namely, we find that our congruent cues lead to more accurate and/or faster detection of coherent PLFs, but also slow down and/or impede the correct rejection (higher FA rate) of scrambled PLFs (details below). In line with the arguments posited by the label-feedback hypothesis and our understanding of the mechanism underlying such hypothesis described in the Introduction, this bias was interpreted as mainly conceptual and perceptual in nature. While we do not entirely exclude the possibility that decision making processes might have played a small role in the task performance, our experimental design makes it unlikely that they were the driving mechanism behind our results. In all three experiments, the probability of any cue-target pairing appearance was strictly controlled for, the cue-target manipulation was based on the overlap in features, i.e., it was at the level of the sematic content that was not apparent to the participants and was even detrimental to their performance, and the response window was very limited. These factors minimised the chance that some kind of post-perceptual, performance-optimising strategy could have been employed during the task performance. Additionally, we found no evidence of a button press bias towards coherent PLF response (‘yes’ answer): neither were ‘yes’ responses given more frequently throughout the experiment, nor were the RTs of FAs shorter than those of CRs/hits. This allowed us to exclude the possibility that the lexically induced bias happened at the response level (e.g., due to motor activation of the finger consistent with the ‘yes’ response) and further confirmed our interpretation of the observed bias as happening at the conceptual and perceptual level.

In Experiment 1, the cueing effect on biological motion detection increased gradually with the number of features encoded in and overlapping between cue and target. In Experiment 2, we observed the same pattern of results as in Experiment 1 and further found that incongruent cues lead to interference with PLF detection. In Experiment 3, biological motion cues were found to only influence PLF orientation discrimination when the PLF had both biological kinematics and form encoded, i.e., with naturally moving PLFs. This finding confirmed that the availability of the form-feature alone encoded in the visual target is insufficient for the lexical cueing effect in this study to be observed.

We found linguistic influence on biological motion perception across tasks, both in biological motion detection and figure orientation discrimination. Figure orientation discrimination does not require the detection or integration of biological kinematics features, but rather puts emphasis on the figure outline, making it a task indirectly related to the kinematics feature encoding. Therefore, these findings suggest that lexically mediated action-relevant features are conceptually and perceptually activated even when the task itself does not directly require their involvement. This in turn supports the claim that lexically mediated activation of category-relevant features occurs automatically, regardless of task^5^. However, the cueing effect was weaker in the orientation discrimination task, indicating that the perceptual task directly related to the diagnostic features of the stimuli enhances the cueing effect (see^18,41–43^).

The compositional nature of the perception of biological motion in this study allowed us to examine how an overlap in the number of features expressed by labels and visual targets affects the strength of a lexical cueing effect on perception. This is particularly relevant because the uniquely powerful influence of lexical cues on perception has been attributed to their ability to activate the neurons coding for the diagnostic features of a labelled category, thereby biasing the perception of visual stimuli towards that category^7,44^. Our findings extend the evidence that feature activation plays an important role in the mechanism driving language-perception interaction by showing that the magnitude of both facilitatory when congruent and detrimental when incongruent cueing effects grows with the number of diagnostic features encoded in the lexical cue and the visual target. This argument is supported by the finding that even underspecified and seemingly unrelated, biological form cues, overlapping with the target on a single (form) feature, affected the speed of the PLF detection in Experiment 1, albeit less strongly than cues with multiple feature overlap (i.e., biological motion words), showing that lexical cueing is not an all-or-nothing phenomenon.

Further, the findings in Experiment 3 show that only when the kinematics feature was encoded in the visual stimuli (i.e., naturally moving PLFs), was target perception susceptible to the influence of motion labels. Conversely, when this information was removed from the visual targets (i.e., gliding figures), thus placing the emphasis on the outline (form) alone, the cueing effect disappeared, even though the discrimination performance was similar to that with naturally moving PLFs. This finding suggests that while lexical effects on perception can be observed even with underspecified (single-feature overlap) lexical cues (see above), they cannot survive impoverished visual target stripped of one of the category-defining features.

Existing work indicates that labels can penetrate the perceptual system and modulate its activation^9,12,14^. These studies, however, have not looked at different aspects of perceptual processing as corresponding to the diagnostic features encoded by the labels. The current study makes that possible by using *compositional* visual targets, i.e., the form of a PLF target is an emergent, global feature, the detection of which is conditional upon a successful local, kinematics-driven configuration^21,23^. We found that biological form cues, targeting the form encoding aspect of PLF detection, exerted a weaker top-down influence on biological motion perception than biological motion cues, targeting both the kinematics and form encoding aspects of PLF detection. This finding suggests that the combined lexical encoding of form and kinematic features biases perceptual activation towards the cued category more comprehensively than form features alone. This in turn illustrates that when it comes to lexical influence on perception, the final percept is guided by interactive yet distinct, feature encoding aspects of that influence.

Importantly, we show that the perceptual bias evoked by linguistic labels is robust enough to mislead participants into reporting an erroneous percept, thus supporting previous findings of detrimental effects of incongruent linguistic cues on visual perception^9^. In our study, this effect was exemplified by the finding that scrambled PLFs were perceived as coherent when cued by congruent biological motion cues, whereas incongruent cues impaired coherent PLF detection. These findings suggest that linguistic labels are strong enough to activate a misleading visual template for PLF configuration, leading to an incorrect report when the lexically labelled action does not match that performed by the PLF. This finding is particularly compelling given that we ensured that our participants detected the PLF stimuli with very high accuracy (> 75%; anything above 55.1% would be significantly above chance level), showing that the perception of even highly detectable targets is still susceptible to lexical cueing.

In conclusion, this study furthers our understanding of language-perception interaction by empirically attesting feature encoding and overlap as the driving mechanism behind linguistic influence on perception, specifically a lexical cueing effect on biological motion perception. We show that linguistic influence can bias conceptual and perceptual processing towards the diagnostic features of the category conveyed through the linguistic input, even at high levels of detection accuracy. Crucially, we show that this linguistically mediated perceptual pre-activation of category-diagnostic features occurs in an involuntary (i.e., automatic) fashion, irrespective of task demands, whether or not the linguistic information is ultimately beneficial to task performance.

## Supplementary Information

Supplementary Information.

## Data Availability

Data collected for this study are publicly available and can be found at https://osf.io/wcvk6. Any other information can be provided upon reasonable request.
