# Supplementary material for: Linguistic labels cue biological motion perception and misperception
Source: Sci Rep. 2021 Aug 26;11:17239. doi: 10.1038/s41598-021-96649-1 (PMC8390742; doi:10.1038/s41598-021-96649-1)
Supplement: Supplementary file 1 — Supplementary Information. [file 41598_2021_96649_MOESM1_ESM.pdf]

Linguistic labels cue biological motion perception and misperception

Supplementary material

Table S1.
Lexical cues

Biological form	Biological motion	General motion
vader *(father)*	houthakker *(woodcutter)*	hagel *(hail)*
broer *(brother)*	wandelaar *(walker)*	sneeuw *(snow)*
oom *(uncle)*	roeier *(rower)*	rook *(smoke)*
echtgenoot *(husband)*	danser *(dancer)*	stoom *(steam)*

The cues were presented in Dutch. English translation is provided in the brackets. The baseline, non-linguistic cue was '####'. Biological motion cues in Experiment 1 were always congruent with the PLF target. In Experiment 2 and 3, they were either congruent or incongruent with the target. General motion cues were always directionally congruent with the RDM mask.

Table S2.
D' and Criterion scores for Experiment 1 and 2

**Experiment 1**			**Experiment 2**		
**Cue categories**	**d'**	**Criterion**	**Cue categories**	**d'**	**Criterion**
Biological motion	2.45	-0.05	Congruent biological motion	2.06	0.04
Biological form	2.49	0.22	Incongruent biological motion	2.04	0.15
General motion	2.51	0.23	General motion	2.10	0.16
No language	2.52	0.21	No language	2.02	0.09

Table S3.
D' and Criterion scores for Experiment 3 (naturals and gliders)

	**Naturals**		**Gliders**	
**Cue categories**	**d'**	**Criterion**	**d'**	**Criterion**
Congruent biological motion	2.27	-0.26	2.86	0.06
Incongruent biological motion	2.32	-0.23	2.77	0.08
General motion	2.29	-0.25	2.76	0.05
No language	2.32	-0.27	2.78	0.09

Posterior densities for the accuracy and RT models in Experiment 1, 2 and 3

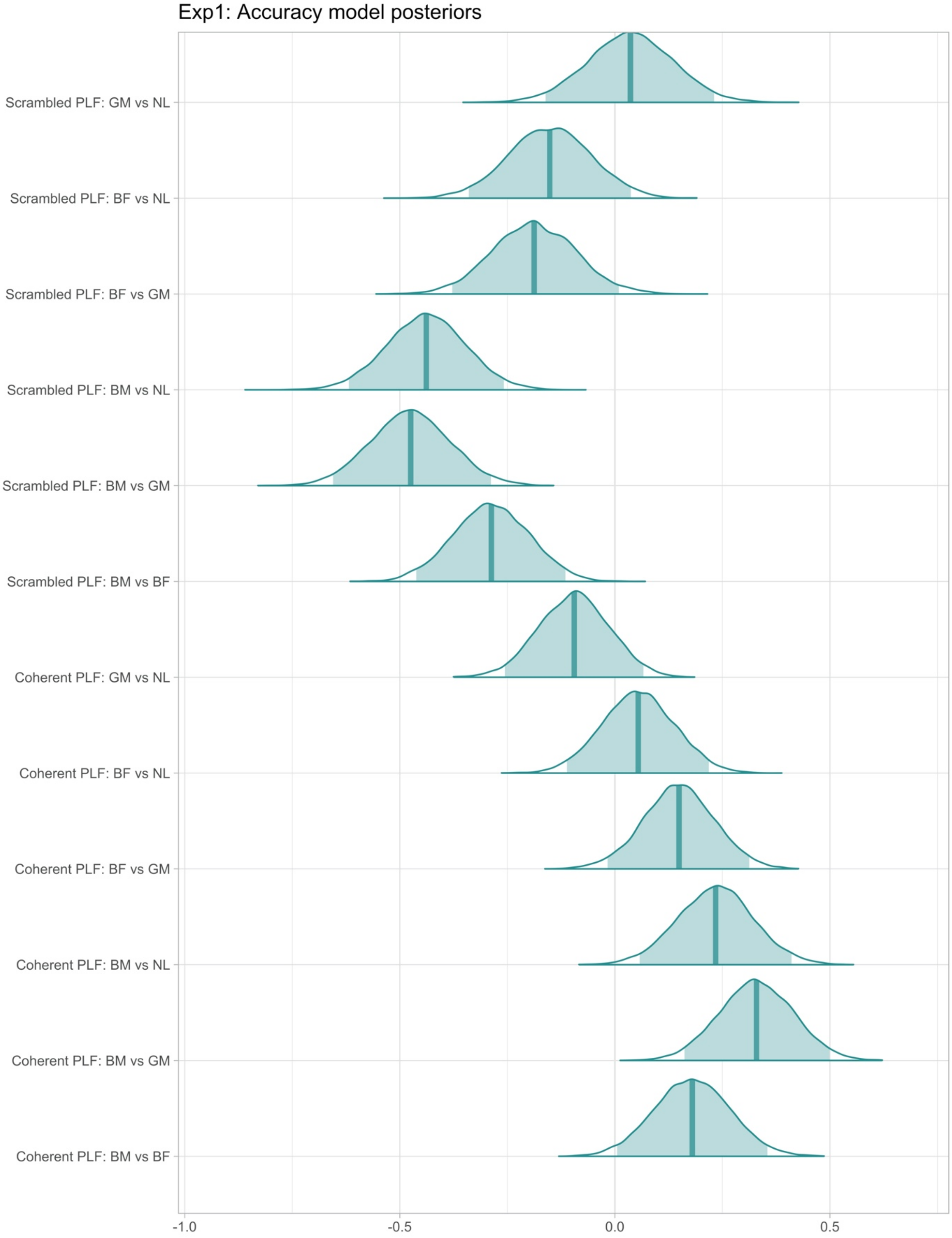

Fig. S1.
Posterior densities for the accuracy model for Experiment 1. Areas filled in green mark 95% credible intervals. Vertical mark is the mean of the distribution. Cue names are as follows: BM – biological motion, BF – biological form, GM – general motion, NL – no language.

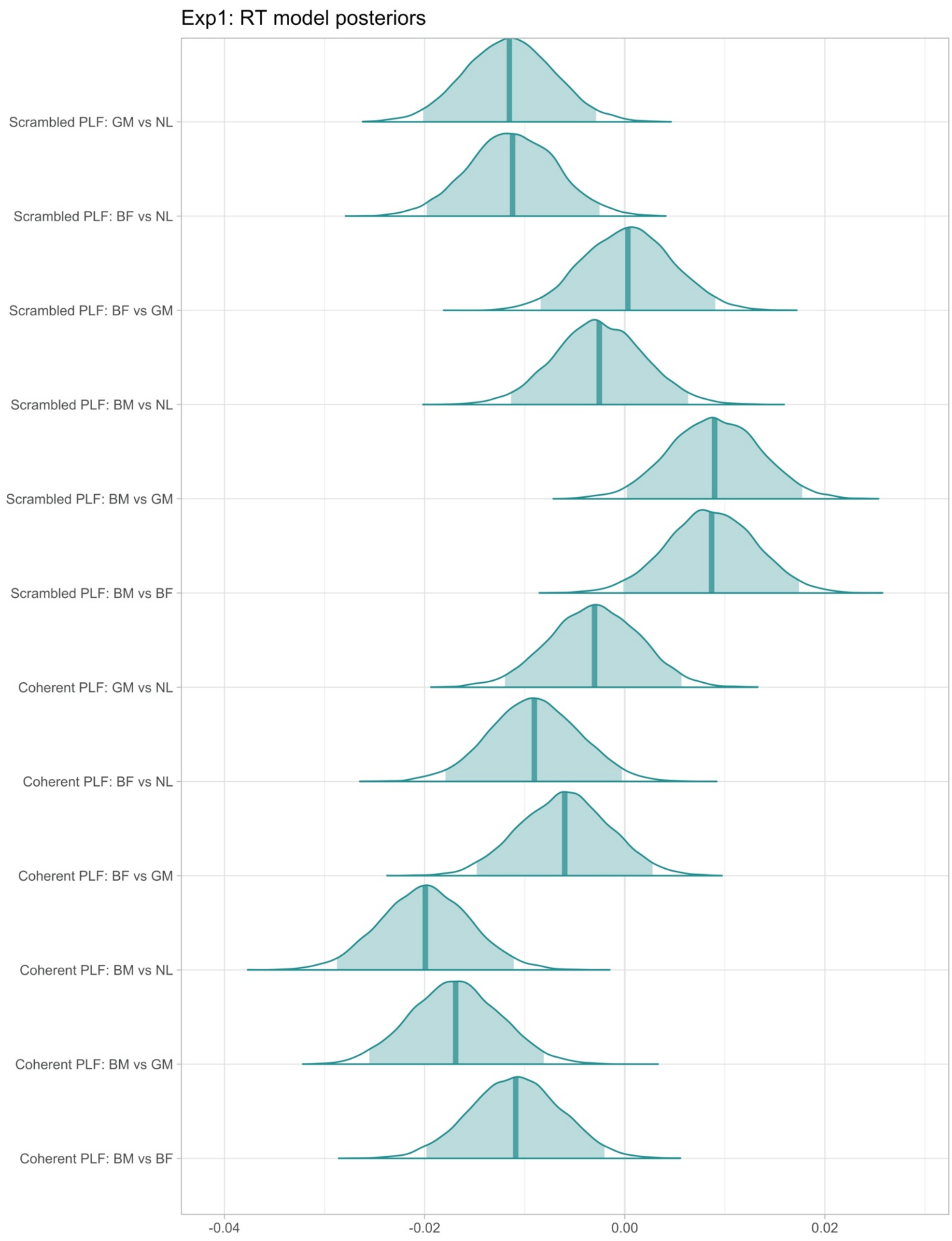

Fig. S2.
Posterior densities for the RT model for Experiment 1. Areas filled in green mark 95% credible intervals. Vertical mark is the mean of the distribution. Cue names are as follows: BM – biological motion, BF – biological form, GM – general motion, NL – no language.

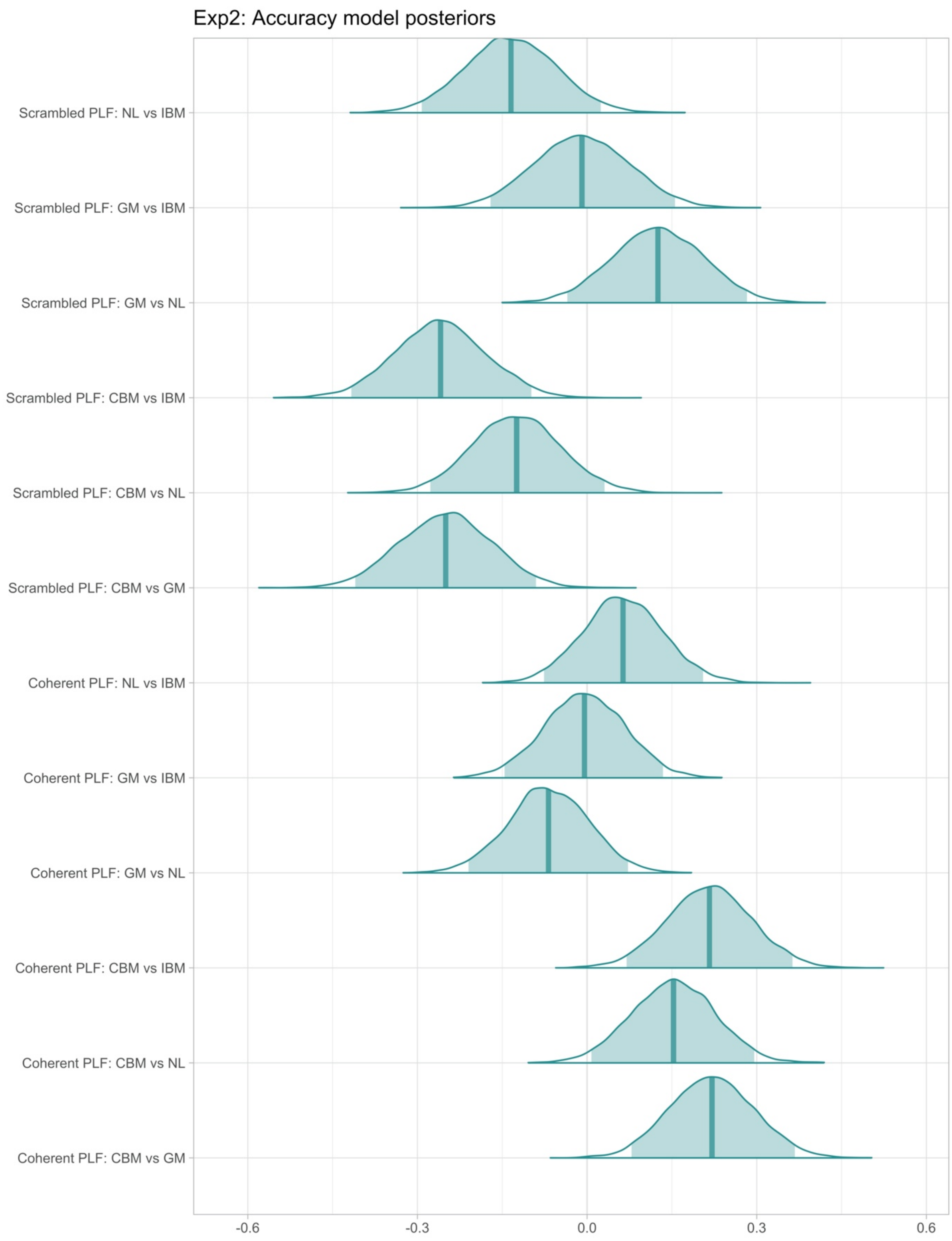

Fig. S3.

Posterior densities for the accuracy model for Experiment 2. Areas filled in green mark 95% credible intervals. Vertical mark is the mean of the distribution. Cue names are as follows: CBM – congruent biological motion, IBM – incongruent biological motion, GM – general motion, NL – no language.

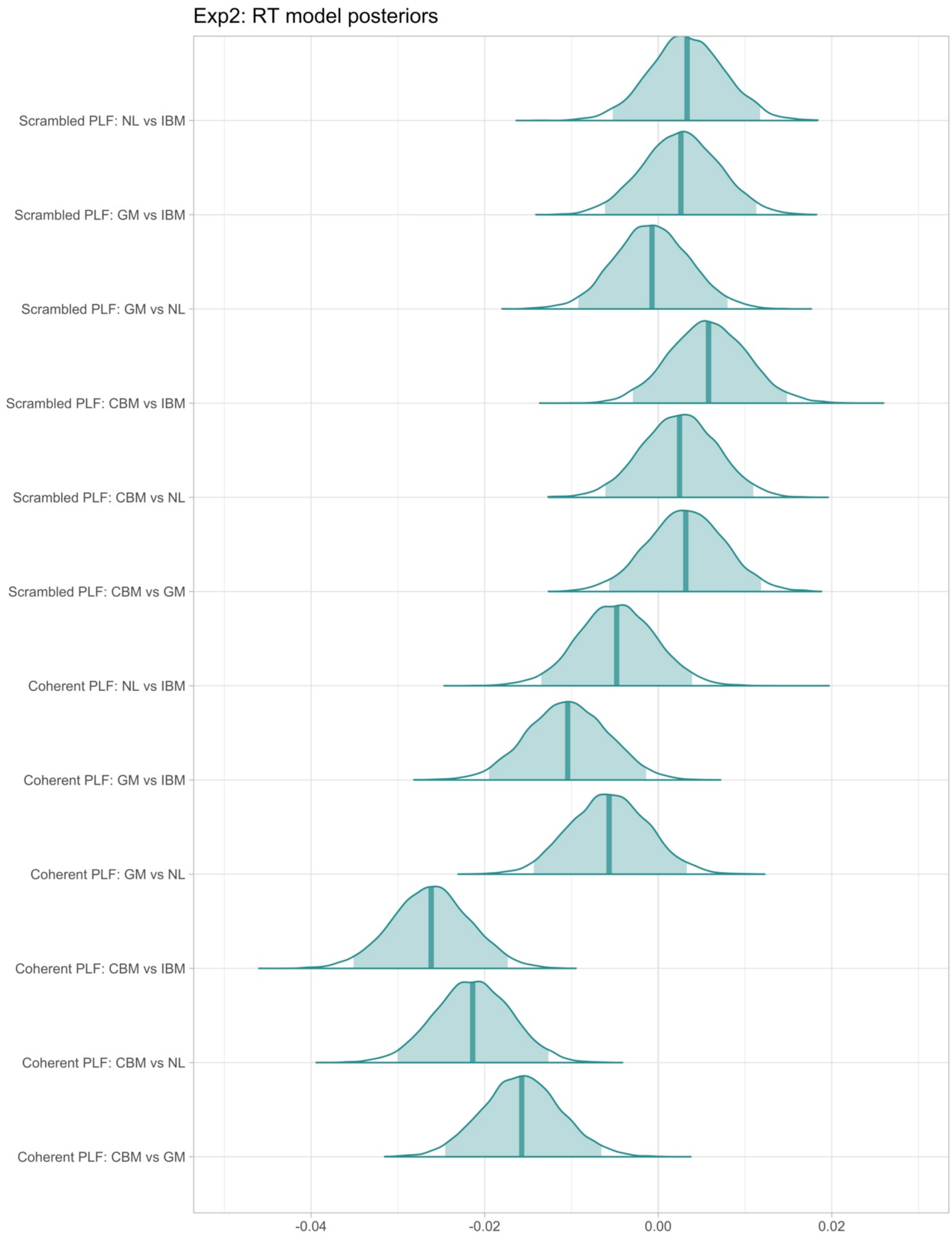

Fig. S4.
Posterior densities for the RT model for Experiment 2. Areas filled in green mark 95% credible intervals. Vertical mark is the mean of the distribution. Cue names are as follows: CBM – congruent biological motion, IBM – incongruent biological motion, GM – general motion, NL – no language.

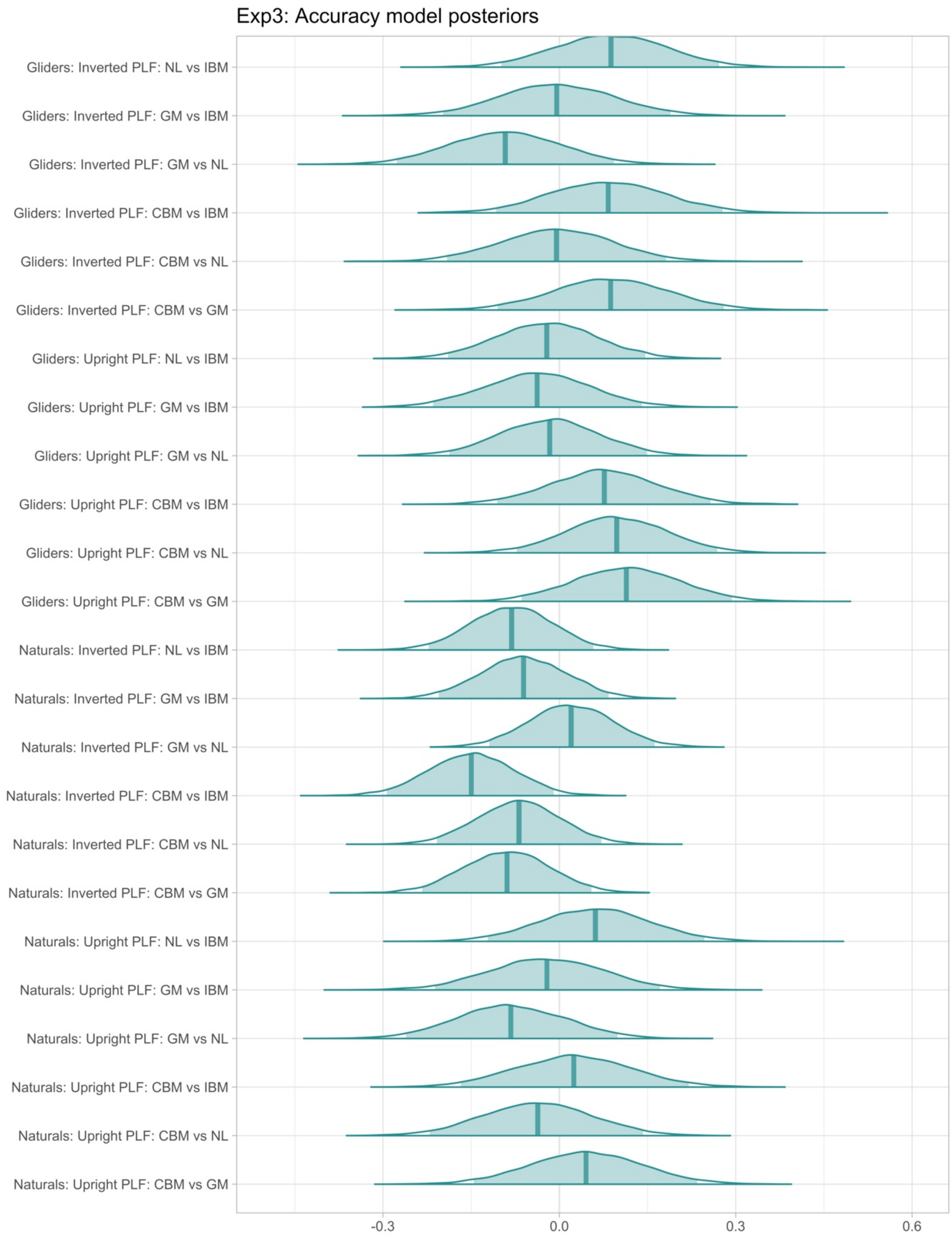

Fig. S5.

Posterior densities for the RT model for Experiment 3. Areas filled in green mark 95% credible intervals. Vertical mark is the mean of the distribution. Cue names are as follows: CBM – congruent biological motion, IBM – incongruent biological motion, GM – general motion, NL – no language.

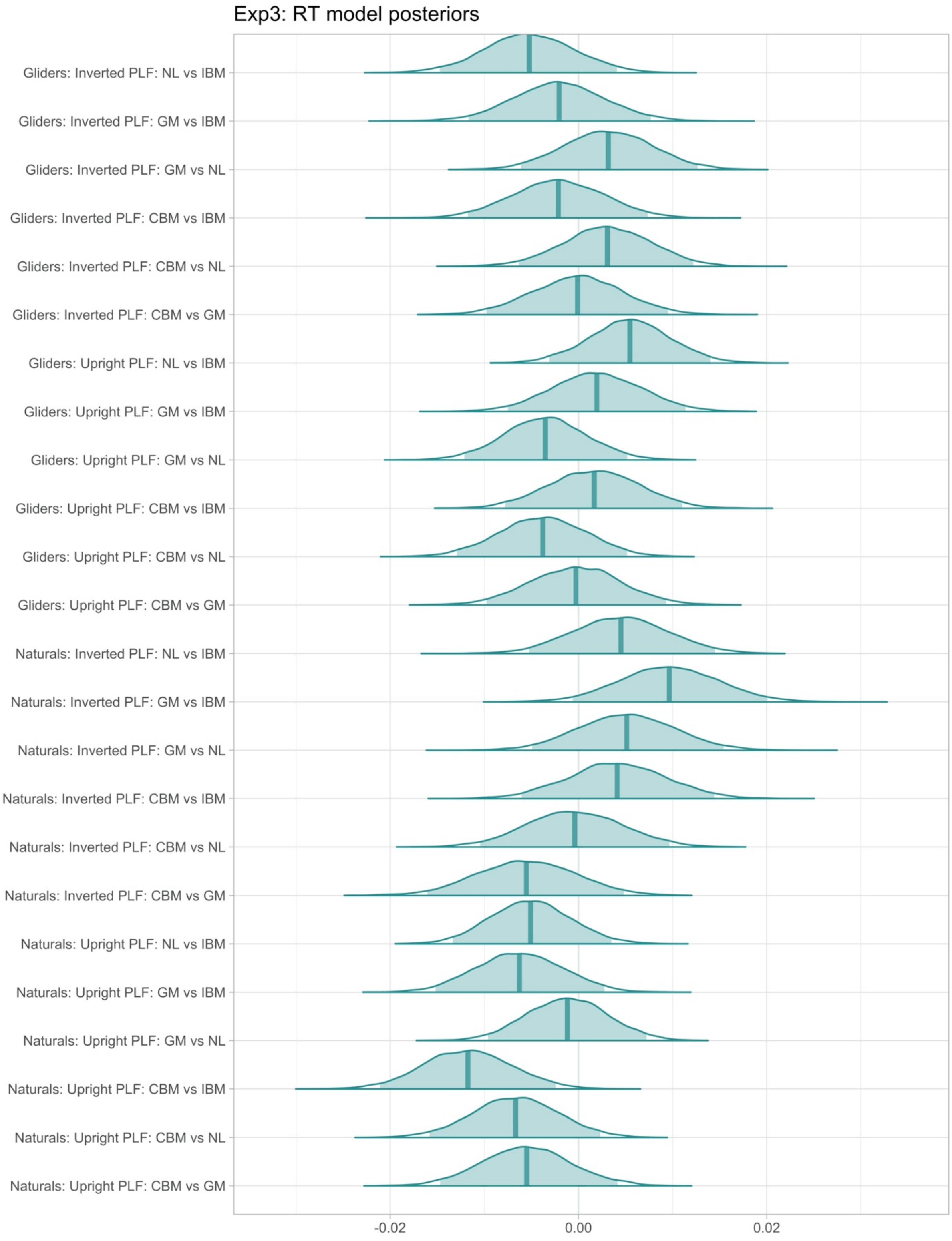

Fig. S6.
Posterior densities for the RT model for Experiment 3. Areas filled in green mark 95% credible intervals. Vertical mark is the mean of the distribution. Cue names are as follows: CBM – congruent biological motion, IBM – incongruent biological motion, GM – general motion, NL – no language.
